# Mechanisms of butylidenephthalide for twitch facilitation in electrically stimulated mouse vas deferens

**DOI:** 10.1080/13880209.2018.1495749

**Published:** 2018-08-19

**Authors:** Chung-Hung Shih, Chi-Ming Chen, Wun-Chang Ko

**Affiliations:** a Department of Internal Medicine, Division of Thoracic Medicine, Taipei Medical University Hospital, Taipei, Taiwan;; b School of Respiratory Therapy, College of Medicine, Taipei Medical University, Taipei, Taiwan;; c Department of Medicinal Chemistry, School of Pharmacy, College of Pharmacy, Taipei Medical University, Taipei, Taiwan;; d Department of Pharmacology, School of Medicine, College of Medicine, Taipei Medical University, Taipei, Taiwan

**Keywords:** Adrenergic prejunctional membrane, potassium channel blocker, voltage-dependent calcium channel blocker

## Abstract

**Context:** The rhizome of *Ligusticum chuaxiong* Hort. (Umbelliferae) has been used by Chinese for several thousand years. Its main constituent, butylidenephthalide (Bdph), was proved to be active in inhibiting rat uterine contractions induced by prostaglandin F_2α_ and was reported to be a nonspecific antispamodic and a blocker of voltage-dependent Ca^2+^ channels (VDCCs).

**Objectives:** The present study investigates the mechanisms of Bdph for twitch facilitation in ICR mouse vas deferens (MVD).

**Materials and methods:** Electrical field stimulation (EFS, supramaximal voltage ranging from 60–90 V, 1 ms, 0.2 Hz) was applied to the isolated MVD in Krebs solution. Interactions between Bdph (50 µM) and calcium antagonist (verapamil, diltiazem or aspaminol) on the EFS-evoked twitch responses were determined. The number of experiments was 3–18.

**Results:** Bdph (50 µM)-induced twitch facilitations from 100 to 391.9% were unrelated to activation of postjunctional cholinergic or adrenergic receptors. Verapamil and Bdph unabolished the twitch facilitation each other. Diltiazem unabolished the Bdph-induced twitch facilitation. In contrast, Bdph abolished those induced by diltiazem. Aspaminol at 20 μM abolished the Bdph-induced twitch facilitation. In contrast, Bdph abolished those induced by aspaminol. Tetraethylammonium and 4-aminopyridine, the K^+^ channel blockers, significantly augmented the Bdph-induced twitch facilitation.

**Discussion and conclusions:** Bdph may bind to the different, more and same subtypes of VDCCs from verapamil, than diltiazem, and as aspaminol does on prejunctional membrane, respectively. Besides a blocker of VDCCs, Bdph may be a blocker of K^+^ channels on prejunctional membrane. Thus, Bdph depolarized the membrane and facilitated the cumulative Ca^2+^-induced twitch responses.

## Introduction

**Figure 1.**
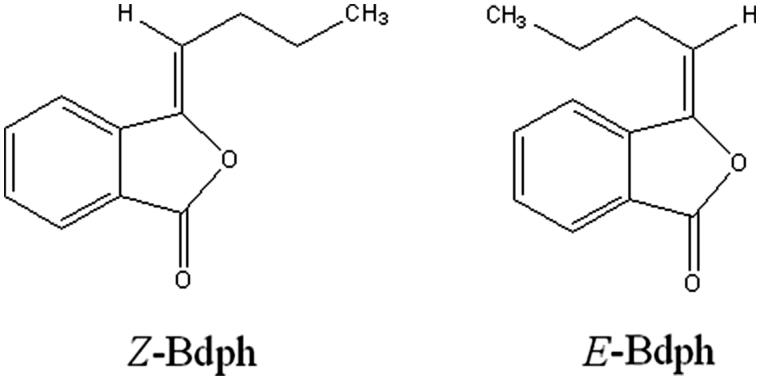
The chemical structure of butylidenephthalide (Bdph; mol. wt. 188.23).

Butylidenephthalide (Bdph, Figure 1), a constituent of the rhizome of *Ligusticum chuanxiong* Hort. (=*L. wallichi* Franch.) (Umbelliferae), has been reported to be a blocker of voltage-dependent Ca^2+^ channels (VDCCs) in guinea-pig ileum (Ko et al. 1997) and in rat aorta (Ko et al. 1998). Bdph has two geometric isomers, *Z*- and *E*-forms. The respective content of *Z*- and *E*-Bdph was reported to be about 85% and 15% in naturally occurring or synthetic product (Ko et al. 1978; Lin et al. 1984). We surprisingly found that both isomers induced twitch facilitation in electrically stimulated mouse vas deferens (MVD) in our preliminary test. Thus, we are interested in investigating the mechanisms of Bdph for twitch facilitation in the animal model.

## Materials and methods

### Drugs and animals

Bdph, a mixture of *Z*- and *E*-Bdph (85% and 15%, respectively), was prepared via a Perkin synthesis as previously described (Lin et al. 1984). 4-Aminopyridine (4-AP, ≥99%), aspaminol (≥98%), atenolol (≥98%), atropine (≥99%), caffeine (99%), diltiazem (≥99%), papaverine (≥98%), pentobarbital (≥98%), phenoxybenzamine (≥97%), prazosin (≥99%), dl-propranolol (≥99%), tetraethylammonium (TEA, ≥98%), tetrodotoxin (≥98%), verapamil (≥99%) and yohimbine (≥98%) were purchased from Sigma-Aldrich Chemical (St. Louis, MO). Other reagents, such as NaCl, KCl, CaCl_2_, NaH_2_PO_4_, NaHCO_3_ and dextrose, were analytical grade.

Male ICR mice weighing 23–25 g were purchased from the Animal Center of the Ministry of Science and Technology (Taipei, Taiwan). The animals were housed in ordinary cages at 22 ± 1 °C with a humidity of 50–60% under a constant 12 h light/dark cycle and provided with food and water *ad libitum*. Under a protocol approved (LAC 74-058) by the Animal Care and Use Committee of Taipei Medical University, the following *in vitro* experiments were performed.

### Mouse vas deferens

After intraperitoneal (i.p.) injection of pentobarbital 50 mg/kg, both sides of MVD were cut under anaesthesia and used. Along these tissues two parallel platinum wires were set and mounted in 7.5 mL of normal Krebs solution for electrical field stimulation (EFS) (Ko 1980). The solution was oxygenated with 95% O_2_–5% CO_2_ and maintained at 37 °C with an initial tension of 0.5 g. The EFS (supramaximal voltage ranging from 60 to 90 V, 1 ms, 0.2 Hz) was generated from Grass S-88 stimulator (Quincy, MA) and supplied to the isolated MVD. The twitch tensions were isometrically recorded on a polygraph (Gould RS3200, Cleveland, OH). The Krebs solution consisted of the following composition (mM): NaCl 119, KCl 4.7, CaCl_2_ 2.5, NaH_2_PO_4_ 1.2, NaHCO_3_ 25 and dextrose 11. The response to EFS was completely abolished by tetrodotoxin (1 μM), suggesting that the response was neurogenic.

### Effects of phenoxybenzamine, adrenergic and cholinergic antagonists on Bdph-induced twitch facilitation

In the absence (control) or presence of phenoxybenzamine (3 or 10 μM, an irreversible nonselective α-adrenoceptor antagonist), prazosin (1 μM, an α_1_-adrenoceptor antagonist), yohimbine (1 μM, an α_2_-adrenoceptor antagonist), dl-propranolol (1 μM, a nonselective β-adrenoceptor antagonist), atenolol (1 μM, an β_1_-adrenoceptor antagonist) or atropine (3 μM, a cholinergic antagonist) for 2 min, Bdph (5–100 μM) or verapamil (1–30 μM), a reference drug, was cumulatively added into normal Krebs solution. The baseline twitch amplitude before adding Bdph or verapamil was taken as 100%. Thus, log concentration–response curves of Bdph or verapamil were constructed.

### Interaction between Ca^2+^ antagonists and Bdph on twitch facilitation

After the twitch responses reached constant, Bdph (50 μM) alone was added into normal Krebs solution to record the twitch facilitation. After the twitch facilitation induced by Ca^2+^ antagonists, such as verapamil (10 μM), diltiazem (20 or 40 μM) and aspaminol (10 or 20 μM), reached constant, Bdph (50 μM) was added into normal or high Ca^2+^ (5 mM) Krebs solution. Similarly, after the twitch facilitation induced by Bdph (25 or 50 μM) reached constant, diltiazem (40 μM) or aspaminol (20 μM) was added into normal or high Ca^2+^ (5 mM) Krebs solution.

### Effects of K^+^ channel blockers on Bdph-induced twitch facilitation

In the absence (control) or presence of TEA (10 or 100 μM) or 4-AP (10 μM) for 2 min, Bdph (5–100 μM) or verapamil (1–30 μM), a reference drug, was cumulatively added into normal Krebs solution. The baseline twitch amplitude before adding Bdph or verapamil was taken as 100%. Thus, log concentration– response curves of Bdph or verapamil were constructed.

### Effects of Bdph and reference drugs on cumulative Ca^2+^-induced twitch responses

In the absence (control) or presence of Bdph (50 μM), phenoxybenzamine (25 μM), diltiazem (40 μM), aspaminol (20 μM), verapamil (10 μM), caffeine (100 μM) or papaverine (10 μM) for 2 min, cumulative Ca^2+^ was added into Ca^2+^-free Krebs solution which omitted CaCl_2_ from normal Krebs solution. The maximal twitch amplitude of control was taken as 100%. Thus, log concentration–response curves of Ca^2+^ were constructed.

### Statistical analysis

All values were expressed as mean ± SEM, *n* was the number of experiments. The difference between two values was determined by Student's *t*-test. Differences of *p* < 0.05 were considered statistically significant.

## Results

### Effects of phenoxybenzamine, adrenergic and cholinergic antagonists on Bdph-induced twitch facilitation

**Figure 2.**
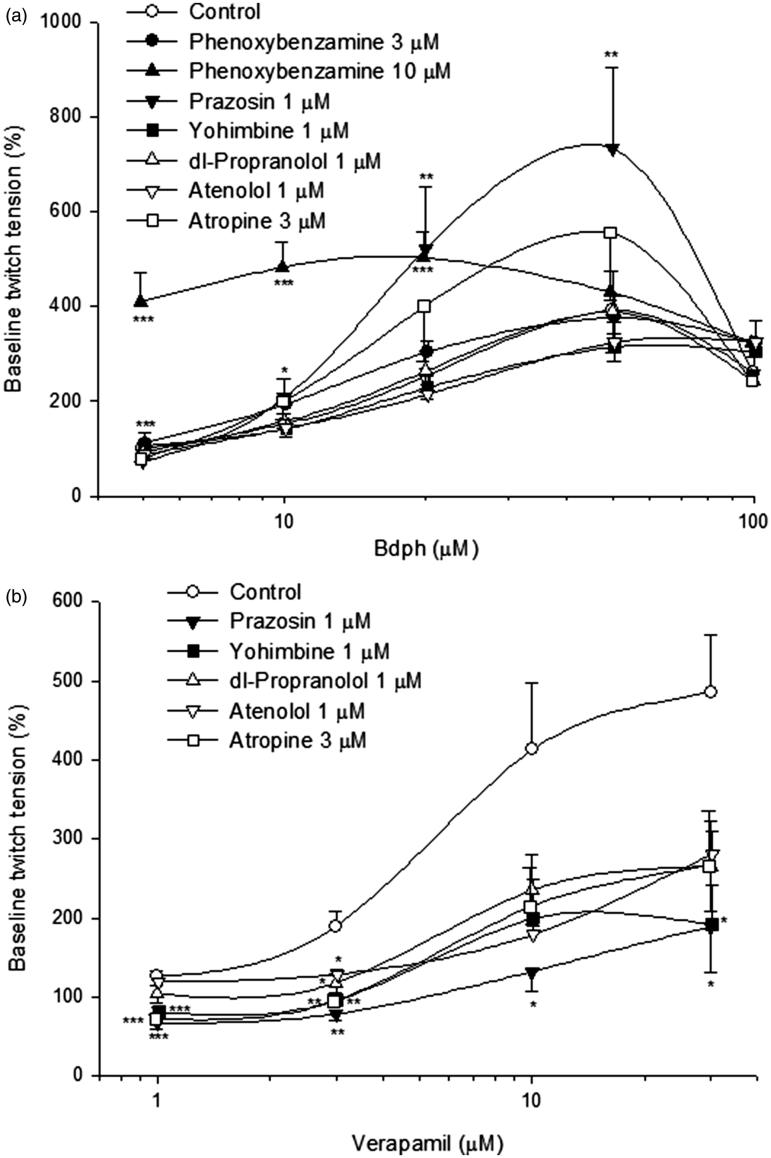
Effects of adrenergic or cholinergic receptor antagonists on log concentration twitch response curves of butylidenephthalide (Bdph, a) and verapamil (b) in electrically stimulated mouse vas deferens. Each point represents mean ± SEM (*n*). The number of experiments (*n*) for Bdph and verapamil was 18 and 20, respectively, whereas those for antagonists were 6–10. *p < 0.05, **p < 0.01, ***p < 0.001 when compared to the control.

The baseline twitch tension for Bdph was 103.2 ± 12.4 mg (*n* = 18), taken as 100%. Bdph concentration dependently facilitated the twitch amplitude to the maximum 391.9 ± 32.6% (*n* = 18) at 50 μM. Phenoxybenzamine at 10, but not 3, μM significantly augmented the twitch facilitations induced by Bdph 5–20 μM. Similarly, prazosin at 1 μM did those induced by Bdph 5–50 μM (Figure 2(a)). However, other antagonists did not influence the log concentration–twitch facilitation curves of Bdph (Figure 2(a)). The baseline twitch tension for verapamil was 104.2 ± 18.4 mg (*n* = 20), taken as 100%. Verapamil concentration dependently facilitated the twitch amplitude to the maximum, 485.4 ± 72.2% (*n* = 20), at 30 μM. All antagonists significantly inhibited the twitch facilitations induced by verapamil at 1 and 3 μM, with the exception of dl-propranolol and atenolol did not influence that by verapamil at 1 μM. Moreover, prazosin significantly inhibited those by verapamil at 10 and 30 μM, but yohimbine did that only at verapamil 30 μM (Figure 2(b)).

### Interaction between Ca^2+^ antagonists and Bdph on twitch facilitation

**Figure 3.**
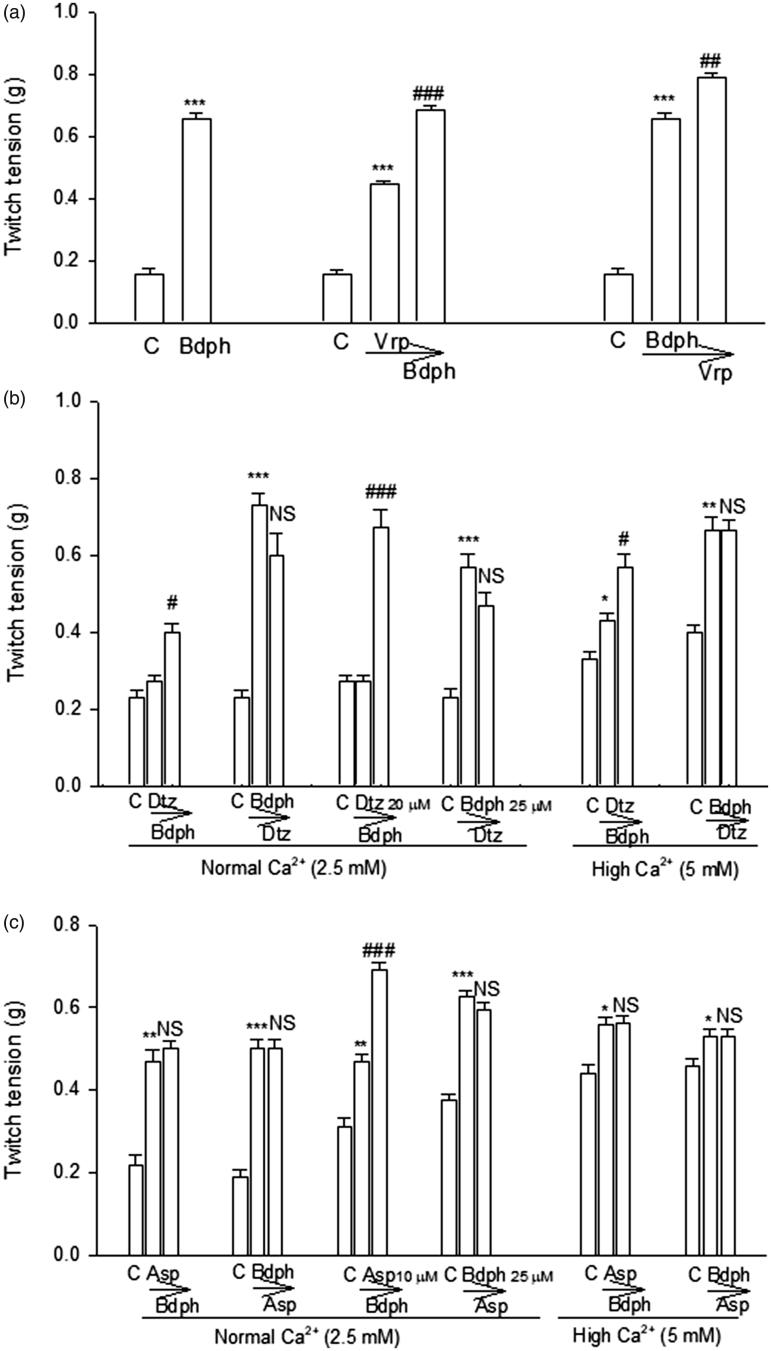
Interaction between butylidenephthalide (Bdph) and Ca^2+^ antagonists on twitch facilitation in electrically stimulated mouse vas deferens. (a) Bdph (50 µM) and verapamil (10 µM) did not abolish their twitch facilitations each other. (b) Preincubation of diltiazem (Dtz, 20 and 40 µM) also did not abolish Bdph (50 µM)-evoked twitch facilitation in normal and high Ca^2+^ (5 mM) Krebs solution. However, preincubation of Bdph (25 or 50 µM) abolished Dtz (40 µM)-evoked twitch facilitation in normal and high Ca^2+^ (5 mM) Krebs solution. (c) Preincubation of aspaminol (Asp, 20 µM) or Bdph (50 µM) abolished their twitch facilitations each other in normal and high Ca^2+^ (5 mM) Krebs solution. However, the preincubation of Asp (10 µM) did not abolish the twitch facilitation evoked by Bdph (50 µM). In contrast, the preincubation of Bdph (25 µM) abolished the twitch facilitation evoked by Asp (20 µM) in normal Krebs solution (c). The number of experiments was 3. *p < 0.05, **p < 0.01, ***p < 0.001 when compared to their control (C). ^#^*p* < 0.05, ^##^*p* < 0.01, ^###^*p* < 0.001 or non-significant (NS) when compared to that of preincubated drug, indicated by arrow.

Verapamil (10 μM) and Bdph (50 μM) did not abolish the twitch facilitation each other in normal Krebs solution (Figure 3(a)), suggesting that Bdph may bind to different subtypes of VDCCs on prejunctional membrane of adrenergic nerve ending from verapamil does. Diltiazem (20 or 40 μM) did not abolish the Bdph (50 μM)-induced twitch facilitations in normal or high Ca^2+^ (5 mM) Krebs solution (Figure 3(b)). In contrast, Bdph (25 or 50 μM) abolished those induced by diltiazem (40 μM) in normal or high Ca^2+^ (5 mM) Krebs solution (Figure 3(b)), suggesting that Bdph may bind to more subtypes of VDCCs on prejunctional membrane of adrenergic nerve ending than diltiazem does. Aspaminol at 20, but not 10 μM abolished the Bdph (50 μM)-induced twitch facilitations in normal or high Ca^2+^ (5 mM) Krebs solution (Figure 3(c)). In contrast, Bdph at 25 or 50 μM abolished those induced by aspaminol (20 μM) in normal or high Ca^2+^ (5 mM) Krebs solution (Figure 3(c)), suggesting that Bdph may bind to the same subtypes of VDCCs on prejunctional membrane of adrenergic nerve ending as aspaminol does.

### Effects of K^+^ channel blockers on Bdph-induced twitch facilitation

**Figure 4.**
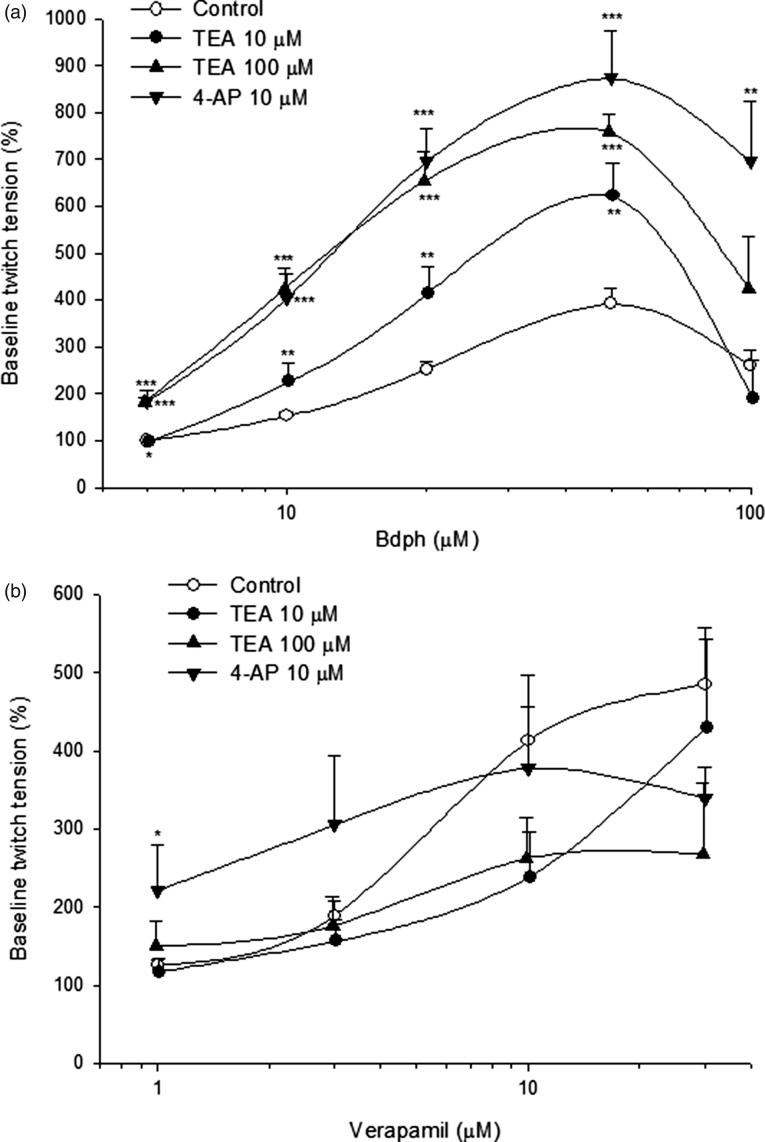
The effects of tetraethylammonium (TEA) and 4-aminopyridine (4-AP) on the log concentration-twitch response curves of butylidenephthalide (Bdph, a) and verapamil (b) in electrically stimulated mouse vas deferens. Each point represents mean ± SEM (*n*). The number (*n*) of experiments for Bdph and verapamil was 18 and 20, respectively, whereas those for antagonists were 6–10. *p < 0.05, **p < 0.01, ***p < 0.001 when compared to the control.

The K^+^ channel blockers, such as TEA (10 and 100 μM) and 4-AP (10 μM), significantly augmented the twitch facilitation induced by Bdph (5–50 μM). Furthermore, 4-AP (10 μM) did that induced by Bdph (100 μM) (Figure 4(a)). Whereas the K^+^ channel blockers did not significantly augment the twitch facilitation induced by verapamil, with the exception of 4-AP did that by verapamil 1 μM (Figure 4(b)).

### Effects of Bdph, phenoxybenzamine, Ca^2+^ channel blockers and phosphodiesterase inhibitors on cumulative Ca^2+^-induced twitch responses

**Figure 5.**
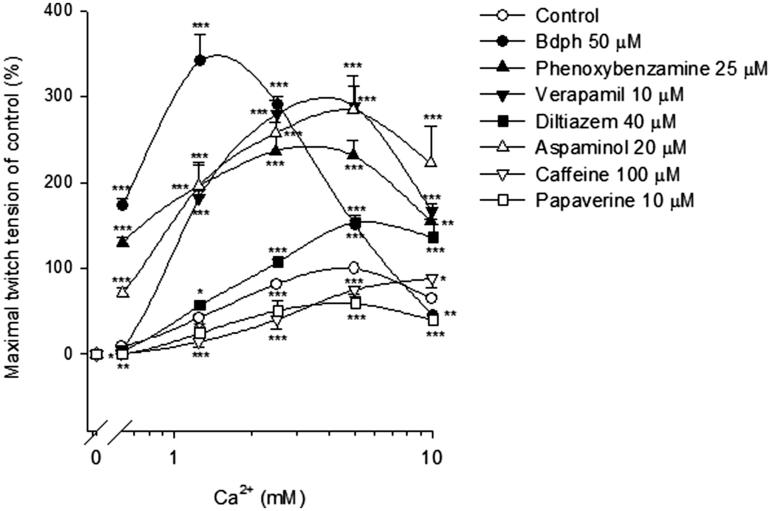
The effects of butylidenephthalide (Bdph), phenoxybenzamine, Ca^2+^ channel blockers and phosphodiesterase inhibitors on the log concentration-twitch response of cumulative Ca^2+^ in electrically stimulated mouse vas deferens. Each point represents mean ± SEM (*n*). The number (*n*) of experiments for control was 15, whereas those for Bdph, phenoxybenzamine, Ca^2+^ channel blockers and phosphodiesterase inhibitors were 6. *p < 0.05, **p < 0.01, ***p < 0.001 when compared to the control.

Cumulative Ca^2+^ concentration dependently increased the twitch tension. The maximal twitch tension induced by Ca^2+^ at 5 mM was 167.3 ± 12.7 mg (*n* = 15), taken as 100%. Bdph (50 μM) significantly augmented the cumulative Ca^2+^ (0.625–5 mM)-induced twitch responses. Similarly, phenoxybenzamine, verapamil, diltiazem and aspaminol also significantly augmented the cumulative Ca^2+^ (0.625–10 mM)-induced twitch responses. In contrast, caffeine and papaverine, the phosphodiesterase (PDE) inhibitors, significantly inhibited the cumulative Ca^2+^ (0.625–10 mM)-induced twitch responses, with the exception that caffeine augmented the twitch response induced by Ca^2+^ at 10 mM (Figure 5).

## Discussion

By EFS, neurotransmitters are released from nerve endings after Ca^2+^ influx from the extracellular space when nerve terminals depolarize and open VDCCs (Bertolino and Llinas 1992; Llinas et al. 1992; Zucker 1993; Wright and Angus 1996; Borderies et al. 1997; Waterman 1997). The pre-junctional VDCCs were reported to consist of L-, N-, P-, Q-, R- and T-types (Zhang et al. 1993; Olivera et al. 1994). N-type Ca^2+^ channels control the release of noradrenaline (NA) from adrenergic nerves (Hirning et al. 1988; Hong et al. 1996) and of acetylcholine (ACh) from cholinergic nerves (De et al. 1990; Boot 1994; Hong et al. 1996). EFS of MVD results in a contraction with two distinct components. The twitch or phasic component is transient and insensitive to nifedipine, an L-type Ca^2+^ channel blocker, whereas the secondary tonic component is sustained for the duration of stimulation and sensitive to nifedipine (Swedin 1971; Kaplita and Triggle 1983). The neurogenic transmitters are NA and adenosine triphosphate (ATP), a co-transmitter of NA, first confirmed to be purine (Westfall et al. 1978). In general, it is considered that the released NA acts on postjunctional α_1_-adrenoceptor to induce nifedipine-insensitive twitch contraction (Minneman et al. 1988), and ATP acts on ligand-gated P2X_1_-receptors to evoke a contraction (Liang et al. 2000; Mulryan et al. 2000), which has been shown to be blocked by nifedipine (Cleary et al. 2003).

In the present results, Bdph-induced twitch facilitations were not influenced by atropine, dl-propranolol, atenolol, yohimbine and phenoxybenzamine (3 μM), suggesting that Bdph-induced twitch facilitations were unrelated to activation of postjunctional cholinergic or adrenergic receptors. Recently, we reported Bdph similar to 4-AP, a blocker of K_v_1 family of K^+^ channels, to antagonize cromakalim, an ATP-dependent K^+^ channel opener, in guinea-pig trachea (Hsu et al. 2014). Thus, Bdph may result in depolarization on the prejunctional membrane of adrenergic nerve ending, and in more releases of NA and ATP. In the present results, at a higher concentration of phenoxybenzamine (10 μM) or prazosin (1 μM) augmented the Bdph-induced twitch facilitations. Phenoxybenzamine was reported to irreversibly block α_1_ and α_2_-adrenoceptors and prazosin has a 1000-fold greater affinity for α_1_- than α_2_-receptors (Williams and Turner 2005). However, both at a higher concentration may block prejunctional α_2_-autoreceptors and results in more NA and ATP release from adrenergic neurons (Cleary et al. 2003; Williams and Turner 2005). In addition, phenoxybenzamine also inhibits NA uptake into both adrenergic nerve terminals and extraneuronal tissues (Williams and Turner 2005). Thus, in this experiment, phenoxybenzamine augmented Bdph-induced twitch facilitations. This augmentation was more obvious than that induced by prazosin at low concentrations of Bdph (Figure 2(a)). In contrast, verapamil-induced twitch facilitations were inhibited by all antagonists at low concentrations, suggesting that these facilitations may be due to endogenous NA and ACh released from adrenergic and cholinergic nerve endings, respectively. Thus, Bdph and verapamil did not influence their facilitations each other (Figure 3(a)). In contrast to MVD, we recently reported Bdph or verapamil (5 µM) inhibited, but not facilitated, the twitch response in electrically stimulated guinea-pig ileum (Chen and Ko 2016). Thus, the facilitation induced by Bdph or verapamil seems dependent on tissue preparation. For example, it has been reported that paradoxical effects of verapamil in vas deferens and adrenal chromaffin cells from which transmitters are released (Bergantin et al. 2013).

In the present results, Bdph may bind to different subtypes of VDCCs on prejunctional membrane from verapamil does. Whereas Bdph may bind to more subtypes of VDCCs on prejunctional membrane than diltiazem does. In contrast, Bdph may bind to the same subtypes of VDCCs on prejunctional membrane as aspaminol does. These Ca^2+^ antagonists are L-type VDCC blockers, but verapamil was also reported to block T-type VDCCs (Bergson et al. 2011). *Z*-Bdph has been reported to inhibit pre-junctional R-type, but not N-type, VDCCs of cholinergic nerve endings (Chen and Ko 2016). Furthermore, TEA (10 or 100 µM) or 4-AP (10 µM) significantly augmented log concentration-facilitation curve of Bdph (Figure 4(a)), but not verapamil with an exception that 4-AP significantly augmented verapamil-induced twitch facilitation at the least concentration of 1 µM (Figure 4(b)). Thus, the mechanisms of Bdph-evoked twitch facilitation may be different from those of verapamil.

Consistently, Bdph, phenoxybenzamine or Ca^2+^ antagonists, such as verapamil, diltiazem and aspaminol, significantly facilitated the cumulative Ca^2+^-induced twitch responses. In contrast, PDE inhibitors, such as caffeine and papaverine, inhibited the twitch responses. It believes that adenosine 3′,5′-cyclic monophosphate (cAMP) may increase after incubation of PDE inhibitors. The increased cAMP may activate cAMP-dependent protein kinase (PKA), and result in increasing calcium extrusion from the intracellular space and uptake to endoplasmic reticula and finally decrease the concentration of intracellular calcium ([Ca^2+^]_i_), thus, inhibit cumulative Ca^2+^-induced twitch responses.

## Conclusions

Bdph-induced twitch facilitations in electrically stimulated MVD were unrelated to activation of postjunctional cholinergic or adrenergic receptors. Bdph may bind to the different, more and same subtypes of VDCCs from verapamil, than diltiazem, and as aspaminol, respectively, does on prejunctional membrane to release more transmitters. Besides a VDCC blocker, Bdph may be a K^+^ channel blocker on adrenergic prejunctional membrane. Thus, Bdph facilitated the twitch responses.
